# Differences in intention to receive clinician-collected and self-collected samples for HPV DNA testing and its determinants between heterosexual males and females in Hong Kong, China: findings of a territory-wide household survey

**DOI:** 10.1186/s12916-024-03788-z

**Published:** 2024-12-02

**Authors:** Zixin Wang, Siyu Chen, Ngai Sze Wong, Annie Wai-Ling Cheung, Zoe Pui-Yee Tam, Sze Long Chung, Denise Pui-Chung Chan, Phoenix K. H. Mo, Eliza Lai-Yi Wong

**Affiliations:** 1 Centre for Health Behaviours Research, JC School of Public Health and Primary Care, The Chinese University of Hong Kong, Hong Kong SAR, China; 2 Faculty of Medicine, the Stanley Ho Centre for Emerging Infectious Diseases, The Chinese University of Hong Kong, Hong Kong SAR, China; 3 Faculty of Medicine, the S.H. Ho Research Centre for Infectious Diseases, The Chinese University of Hong Kong, Hong Kong SAR, China; 4 Centre for Health Systems and Policy Research, JC School of Public Health and Primary Care, The Chinese University of Hong Kong, Hong Kong SAR, China

**Keywords:** Clinician-collected samples for HPV DNA testing, Self-collected samples for HPV DNA testing, Behavioral intention, Illness representation of sexually transmitted infections, Health Belief Model, Heterosexual males, Females, China

## Abstract

**Background:**

Human papillomavirus (HPV) infection is prevalent among people who are sexually active. This study aimed to compare the levels of behavioral intention to receive free clinician-collected and self-collected samples for HPV DNA testing and its determinants between heterosexual males and females in Hong Kong, China.

**Methods:**

This is a secondary analysis of a territory-wide survey conducted in Hong Kong between May 2021 and March 2022. Participants were sexually active adult Hong Kong residents who were able to communicate in English or Chinese. Invitation letters were mailed to residential addresses that were geographically randomly selected. All sexually active adult household members were invited to complete a self-administered online survey. Multivariate logistic regression analyses were fitted.

**Results:**

Out of 45,394 invitations, 1265 surveys were collected, and 487 heterosexual males and 741 females were included in the analysis. More females than heterosexual males intended to take up free clinician-collected samples for HPV DNA testing (76.7% versus 62.2%, *p* < 0.001). Similar proportion of heterosexual males and females intended to receive free self-collected samples for HPV DNA testing (67.8% versus 72.6%, *p* = 0.20). Perceived existing treatment could control sexually transmitted infections (STI) (treatment control), more concerned about STI (concern), perceived more benefits, cue to action (suggested by significant others), and self-efficacy related to HPV testing were associated with higher intention to receive clinician-collected samples for HPV testing in both groups. Heterosexual males who perceived more severe symptoms if contracted STI (identity), longer duration of STI (timeline), more negative effects of STI on their lives (consequences), more understanding of STI (coherence), and stronger negative emotions if contracted STI (emotions) also had higher behavioral intention to take up clinician-collected samples for HPV testing. In addition, perceived more benefits, cue to action, and self-efficacy related to self-collected samples for HPV DNA testing were associated with behavioral intention to take up such testing in both groups.

**Conclusions:**

HPV DNA testing was under-utilized in Hong Kong. Free self-collected samples for HPV testing were highly acceptable by both heterosexual males and females. Illness representation of STI and the Health Belief Model could explain intentions to take up HPV DNA testing.

## Background

Human papillomavirus (HPV) infection is prevalent among people who are sexually active and causes genital warts and numerous types of cancers (cervical, oropharyngeal, penile, anal, and head and neck cancers) [1]. Cervical cancer is the fourth most common cancer in women globally [2]. China reported the second-highest incidence and mortality rates of cervical cancers globally [3]. In Hong Kong, China, where this study was conducted, Cervical cancer is the seventh commonest cancer among females with an estimated incidence of 8.5/100,000 [4, 5]. All women who have ever had sexual experience should receive regular screening for HPV infection, which can detect high-risk HPV and early abnormal cervical cell changes. Timely treatment of precancerous changes can prevent their progression to cervical cancers. The main purpose of HPV screening among heterosexual males is to protect their female sex partners. Sexual intercourse is a necessary step to acquire HPV infection among HPV-negative women [6]. Heterosexual males’ HPV positivity increases the risk of non-high-risk HPV infection and therefore increases the risk of cervical lesions development in their female sex partners [7]. Moreover, among females with high-grade cervical lesions, their male sex partners could be important hosts and vectors of HPV infection, resulting in looping of transmission, infection, and sero-clearance between the sex partners (e.g., couples). Recurrence of cervical diseases in females could occur [7]. Such risk of heterosexual HPV transmission is high in China, as males have similar prevalence of HPV infection comparing to females (14.5% versus 15.6%) [8]. If a heterosexual male is screened to be HPV positive, he may adopt measures to reduce the risk of transmission to his female sex partners before the virus clearance, such as abstinence, consistent condom use, or taking up HPV vaccination.

Screening of HPV infection can be performed using either cytology testing or HPV DNA testing [9–11]. The cytology testing identifies patients with cellular changes in the epithelial cells, while the DNA testing detects whether the sampled cells have the DNA of high-risk types of HPV. Previous studies supported that HPV DNA testing was more effective than today’s commonly used HPV screening methods [12, 13]. The World Health Organization (WHO) hence recommends the use of HPV DNA testing as a first-choice screening method [14]. In Hong Kong Special Administrative Region (SAR) of China, women aged 30–64 years who ever had sexual experience are recommended to undertake cytology testing every 3 years or HPV DNA testing every 5 years or co-testing (both cytology and DNA testing) every 5 years after two consecutive normal annual screening [11]. Fully subsidized cytology testing and clinician-collected samples for HPV DNA testing are provided by public hospitals and/or social hygiene clinics to females in Hong Kong SAR [15]. Symptomatic males can also receive free cytology testing and clinician-collected samples for HPV DNA testing at social hygiene clinics [15]. The Hong Kong government has been implementing a cervical screening program since 2004, offering cytology testing and clinician-collected samples for HPV DNA testing to females at HK$100–250 (US$12.7–31.8) per person [16]. In addition, males can receive self-financed HPV DNA testing at private clinics and community-based organizations.

Self-collected samples for HPV DNA testing refer to collecting samples by users instead of healthcare providers at home or other private places for HPV testing. Users can send the collected samples to designated laboratories, which will examine the sample and inform them of the testing results [17]. As compared to clinician-collected samples for HPV DNA testing, self-collected samples for HPV testing have similar accuracy but cause less pain and discomfort to the users [18–21]. Self-collected samples for HPV DNA testing have the potential to overcome obstacles to receive HPV testing, and hence increase its coverage [22, 23]. In Hong Kong, a pilot program was implemented by a local university providing fully subsidized self-collected samples for HPV testing services to females [24].

Facilitators and barriers to receive HPV DNA testing among females were extensively studied. In the literature, the uptake rate of any HPV DNA testing ranged from 26.3 to 72.0% among females in different countries [25–29]. Previous studies showed that 45.6–69.7% of females intended to use clinician-collected samples for HPV testing [30, 31], and 58.7–68.2% intended to take up self-collected samples for HPV testing [32, 33]. Determinants of actual uptake or behavioral intention to use clinician-collected or self-collected samples for HPV testing included sociodemographic characteristics, history of HPV infection, sexual behaviors, perceptions of HPV testing and cervical cancers, and perceived stigma originated from service providers [25, 29–31, 34]. Relatively few studies investigated HPV testing utilization among males [35–37], and most of these studies focused on men who have sex with men (MSM) [35, 36]. Our literature review identified one study targeting heterosexual males, which found that fear of genital warts and being able to communicate the HPV test results with sex partners were facilitators of HPV testing uptake [37].

Illness representations refer broadly to how people think about a disease or health condition. The Common Sense Model of Self-regulation suggests that people will construct schematic representations of an illness based on available information [38]. This theoretical model suggests that a health threat (e.g., sexually transmitted infections [STI]) would activate cognitive representations for regulating the objective threat and emotional representations to regulate emotions (e.g., fear, anxiety) [38]. Both cognitive and emotional representations guide the coping strategies to be employed by an individual to deal with such health threat. This model is extended to explain health-related behaviors among healthy people [39, 40]. A previous study showed that illness representation of HPV was associated with HPV screening behaviors among females [41]. Given the susceptibility to genital warts among people in Hong Kong, it is possible that illness representation of STI would also affect people’s decision to use HPV testing. However, no study had tested such hypothesis.

To address these gaps, this study compared behavioral intention to receive free clinician-collected and self-collected samples for HPV DNA testing between heterosexual males and females in Hong Kong, China. In addition, potential factors associated with behavioral intention to receive free clinician-collected and self-collected samples for HPV testing were also explored, including sociodemographic characteristics, illness representation of STI, perceptions of HPV testing in general, and perceptions specific to self-collected HPV testing. We hypothesized that levels of behavioral intention to receive clinician-collected and self-collected samples for HPV testing and their determinants would be different between heterosexual males and females.

## Methods

### Study design

This is a territory-wide survey among adult sexually experienced residents in Hong Kong with a recruitment period between May 2021 and March 2022.

### Participants and data collection

The inclusion criteria for the original household survey included: (1) aged 18 years or above, (2) had ever had sexual behaviors in their lifetime, (3) normally living in Hong Kong, and (4) able to read and write in English or Chinese. Under the sampling frame of residential building groups developed by the Census and Statistics Department of Hong Kong and the Centamap Company Limited, the residential addresses dataset was geographically randomly selected for mailing invitation letters [42]. All eligible household members were invited to join the survey. They could scan a Quick Response (QR) code printed on the invitation letter to complete a self-administered online survey. Online informed consent was sought before starting the survey. Hard copies of the questionnaire were available to eligible participants upon request. Seven participants completed the survey in hard copies. A HK$25 (US$3.2) catering voucher was given to each participant who had completed the survey. A total of 45,394 invitation letters have been sent to selected residential addresses, covering 18 districts. Between 1 May 2021 and 31 March 2022, 1265 surveys were collected. This study focused on heterosexual males and females. Therefore, we excluded 37 MSM in the analysis. Ethical approval was obtained from the Joint Chinese University of Hong Kong – New Territories East Cluster Clinical Research Ethics Committee (reference number: CREC 2020.436).

### Measurements

#### Background characteristics

Information collected included socio-demographic characteristics (age, ethnicity, marital status, education level, employment status, monthly personal income, whether living alone, and having medical insurance), history of chronic diseases, history of HPV infection, genital warts, HPV testing, and HPV vaccination. Participants also reported on sexual behaviors in the past year, including condom use with regular, non-regular, and commercial sex partners. A regular sex partner (RP) was defined as a stable sex relationship such as a spouse or stable boyfriend or girlfriend, while a commercial sex partner was defined as people who exchanged sex explicitly for money or goods. A non-regular sex partner (NRP) was defined as a sex partner who was neither a RP nor a commercial sex partner, mainly referring to a one-night stand. Similar definition of RP, NRP, and commercial sex partner was commonly used in published studies [43].

#### Behavioral intention to take up free clinician-collected and self-collected samples for HPV DNA testing

Participants first read a statement explaining clinician-collected samples for HPV DNA testing (“Doctors can use a swab to collect sample from you and examine whether you are infected with some types of HPV”), and then indicated their likelihood of taking up free clinician-collected HPV testing in the next year (response categories: 1 = very unlikely, 2 = unlikely, 3 = neutral, 4 = likely, and 5 = very likely). Participants read another statement about self-collected samples for HPV DNA testing (“You can collect the sample by yourself at home or other private places using a self-sampling kit, send the sample to designated clinics, who will examine the sample and inform you the results”) before answering another question measuring their likelihood of taking up free self-collected samples for HPV testing in the next year (response categories: 1 = very unlikely, 2 = unlikely, 3 = neutral, 4 = likely, and 5 = very likely). Similar ways to measure behavioral intention to take up clinician-collected and self-collected samples for HPV testing were used in published studies [35]. Participants who answered “likely” or “very likely” to the aforementioned questions were defined as having a behavioral intention. The same definition was used in a number of published studies [35, 44, 45].

#### Illness representation of STI

Brief Illness Perception Questionnaire (BIPQ) was used to measure participants’ illness representation of STI [46]. The tool was modified by asking the participants to imagine a hypothetical scenario of contracting STI and replacing “illness” with “STI” from the original items [46]. A similar approach has been used by numerous studies to assess illness representations among healthy people who are at risk of a disease [39, 40]. An open-ended question about what the participant believes is the cause of illness was excluded from this study. The remaining eight items measured domains including identity (“If you contracted STI, how much will you experience symptoms from the illness?”), timeline (“If you contracted STI, how long do you think your illness will continue?”, personal control (“If you contracted STI, how much control do you feel you have over your illness?”), treatment control (“If you contracted STI, how much do you think existing treatment can help your illness?”), consequences (“If you contracted STI, how much does your illness affect your life?”), concern (“If you contracted STI, how concerned are you about your illness?”), coherence (“If you contracted STI, how well do you feel you understand STI?”), and emotions (“If you contracted STI, how much does your illness affect your emotionally?”). Each item was rated from 0 to 10.

#### Perceptions related to HPV DNA testing in general based on the Health Belief Model (HBM)

Two scales validated in the Chinese population were used to measure perceived benefits and perceived barriers related to HPV DNA testing in general [35]. They were the 2-item Perceived Benefit Scale (e.g., “HPV DNA testing could detect HPV infection earlier, so as to have better treatment outcomes”) and the 2-item Perceived Barrier Scale (e.g., “HPV DNA testing would cause pain and discomfort”). Two validated single items measured perceived cue to action (“People who are important to you would suggest you to take up HPV DNA testing”) and perceived self-efficacy (“It is easy for you to take up HPV DNA testing in the next year if you want to”). The aforementioned items were rated using a 5-point Likert scale from 1 = strongly disagree to 5 = strongly agree. The Cronbach’s alpha for the Perceived Benefit Scale and the Perceived Barrier Scale was 0.69 and 0.73, respectively.

#### Perceptions specific to self-collected samples for HPV DNA testing based on the HBM

We added one new item “Performing self-collected samples for HPV DNA testing could reduce the risk of SARS-CoV-2 infection during the pandemic” to a validated scale measuring perceived benefit specific to self-collected samples for HPV DNA testing [35], and formed the 4-item Perceived Benefit Specific to Self-collected Samples for HPV DNA Testing Scale. The Cronbach’s alpha of this scale was 0.69. One factor was identified by exploratory factor analysis, explaining 55.6% of the total variance. Three validated items measured perceived barrier (“You concern about the accuracy of self-collected samples for HPV DNA testing”), cue to action (“People who are important to you suggest that you take up self-collected samples for HPV DNA testing”), and perceived self-efficacy specific to self-collected samples for HPV DNA testing (“It is easy for you to take up self-collected samples for HPV DNA testing in the next year if you want to”) [35]. The response categories for the abovementioned items were 1 = strongly disagree, 2 = disagree, 3 = neutral, 4 = agree, and 5 = strongly agree.

### Statistical analysis

Descriptive statistics of all studied variables were presented. Differences in background characteristics between heterosexual males and females were compared using Chi-square testing. Between-group differences in the behavioral intention to take up clinician-collected and self-collected samples for HPV DNA testing, BIPQ of STI, and perceptions related to HPV testing in general and specific to self-collected samples for HPV testing were compared using logistic or linear regression models, after controlling for background characteristics with significant between-group differences. The subsequent analysis was performed in each group of participants. Behavioral intention to take up clinician-collected and self-collected samples for HPV DNA testing was used as dependent variables. Bivariate logistic regression models were first fitted to assess the associations between background characteristics and the dependent variables. A single multivariate logistic regression model was then fitted, including all significant background characteristics and one independent variable of interest (i.e., BIPQ of STI and perceptions related to HPV DNA testing) at a time. The crude odds ratios (OR), adjusted odds ratios (AOR), and their 95% confidence interval (CI) were obtained. SPSS 26.0 (IBM Corp., Armonk, NY, USA) was used for data analysis, with *p* < 0.05 as the level of statistical significance.

## Results

### Background characteristics of the participants

Majority of the participants were more than 40 years old (heterosexual males: 67.4%, females: 50.9%), Chinese (heterosexual males: 99.2%, females: 98.4%), married (heterosexual males: 65.7%, females: 61.0%), full-time employed (heterosexual males: 73.9%, females: 61.4%), living with other household members (heterosexual males: 89.9%, females: 92.8%), and having medical insurance (heterosexual males: 69.8%, females: 66.7%). Compared to female participants, heterosexual males were older (*p* < 0.001), and more likely to have full-time employment (*p* < 0.001), a monthly personal income of more than HK$30,000 (US$3847) (38.2% versus 22.5%, *p* < 0.001), any chronic diseases (22.2% versus 12.4%, *p* < 0.001), and history of genital warts (3.7% versus 1.5%, *p* = 0.01). In contrast, more females had history of HPV testing (22.7% versus 5.1%, *p* < 0.001) and HPV vaccination in lifetime (26.2% versus 4.5%, *p* < 0.001) comparing to heterosexual males. In the past year, more heterosexual males had sexual intercourse with NRP (6.2% versus 3.4%, *p* = 0.02) and commercial sex partners (8.2% versus 0.3%, *p* < 0.001) and reported condomless sex with commercial sex partners (2.1% versus 0.3%, *p* = 0.02), as compared to females (Table 1).

**Table 1:** Background characteristics of the participants

	Heterosexual male (*n* = 487)	Female (*n* = 741)	*P* values
	*n* (%)	*n* (%)	
**Socio-demographic characteristics**			
Age group, years			
18–30	47 (9.7)	103 (13.9)	
31–40	112 (23.0)	261 (35.2)	
41–50	104 (21.4)	148 (20.0)	
51–60	128 (26.3)	141 (19.0)	
> 60	96 (19.7)	88 (11.9)	< 0.001
Ethnicity			
Chinese	483 (99.2)	729 (98.4)	
Other ethnicities	4 (0.8)	12 (1.6)	0.23
Marital status			
Currently single/divorced/widowed	167 (34.3)	289 (39.0)	
Married	320 (65.7)	452 (61.0)	0.10
Education level			
Primary or below	13 (2.7)	30 (4.0)	
Secondary	151 (31.0)	258 (34.8)	
Diploma or associate degrees	64 (13.1)	120 (16.2)	
University or above	259 (53.2)	333 (44.9)	0.03
Employment status			
Full time	360 (73.9)	455 (61.4)	
Part-time/self-employed/unemployed/retired/student/housewife	127 (29.3)	286 (38.6)	< 0.001
Monthly personal income, HK$ (US$)			
< 15,000 (1923)	67 (13.8)	166 (22.4)	
15,000–30,000 (1923–3846)	152 (31.2)	216 (29.1)	
30,001–50,000 (3847–6410)	107 (22.0)	107 (14.4)	
> 50,000 (6410)	79 (16.2)	60 (8.1)	
Refuse to disclose	82 (16.8)	192 (25.9)	< 0.001
Living alone			
No	438 (89.9)	688 (92.8)	
Yes	49 (10.1)	53 (7.2)	0.07
Having medical insurance			
No	147 (30.2)	247 (33.3)	
Yes	340 (69.8)	494 (66.7)	0.25
**Health conditions**			
Presence of chronic diseases			
Heart diseases	14 (2.9)	8 (1.1)	0.02
Hypertension	61 (12.5)	46 (6.2)	< 0.001
Diabetes mellitus	31 (6.4)	12 (1.6)	< 0.001
Cancers	4 (0.8)	9 (1.2)	0.51
Hypercholesterolemia	50 (10.3)	39 (5.3)	0.001
Any of above	108 (22.2)	92 (12.4)	< 0.001
History of HPV infection			
No	485 (99.6)	699 (94.3)	
Yes	2 (0.4)	42 (5.7)	< 0.001
History of genital warts			
No	469 (96.3)	730 (98.5)	
Yes	18 (3.7)	11 (1.5)	0.01
**History of HPV testing and HPV vaccination**			
Ever had Pap smear testing experience			
No		188 (25.4)	
Yes	N.A	553 (74.6)	N.A
Use of HPV DNA testing in the lifetime			
No	462 (94.9)	573 (77.3)	
Yes	25 (5.1)	168 (22.7)	< 0.001
History of HPV vaccination			
No	465 (95.5)	547 (73.8)	
Yes	22 (4.5)	194 (26.2)	< 0.001
**Sexual behaviors in the past year**			
Sexual intercourse with regular sex partners (RP)			
No	294 (60.4)	458 (61.8)	
Yes	193 (39.6)	283 (38.2)	0.61
Sexual intercourse with non-regular sex partners (NRP)			
No	457 (93.8)	716 (96.6)	
Yes	30 (6.2)	25 (3.4)	0.02
Sexual intercourse with commercial sex partners			
No	447 (91.8)	739 (99.7)	
Yes	40 (8.2)	2 (0.3)	< 0.001
Condomless sex with RP			
No	334 (68.6)	540 (72.9)	
Yes	153 (31.4)	201 (27.1)	0.10
Condomless sex with NRP			
No	466 (95.7)	721 (97.3)	
Yes	21 (4.3)	20 (2.7)	0.12
Condomless sex with commercial sex partners			
No	477 (97.9)	739 (99.7)	
Yes	10 (2.1)	2 (0.3)	0.02

*N.A.* not applicable

### Behavioral intention to take up free clinician-collected and self-collected samples for HPV DNA testing

As compared to heterosexual males, more females intended to take up free clinician-collected samples for HPV DNA testing in the next year (76.7% versus 62.2%, *p* < 0.001). The between-group difference in the behavioral intention to take up free self-collected samples for HPV DNA testing in the next year was not of statistically significant (heterosexual males: 67.8% versus females: 72.6%, *p* = 0.20) (Table 2).

**Table 2:** Perceptions related to HPV, HPV DNA testing, and sexually transmitted diseases

	Heterosexual male (*n* = 487)	Female (*n* = 741)	Unadjusted *P* values	Adjusted *P* values
	n (%)	n (%)		
**Behavioral intention to take up HPV DNA testing**				
Likelihood to take up free clinician-collected samples for HPV testing in the next year				
No (very unlikely/unlikely/neutral)	184 (37.8)	173 (2.3)		
Yes (likely/very likely)	303 (62.2)	568 (76.7)	< 0.001	< 0.001
Likelihood to take up free self-collected samples for HPV testing in the next year				
No (very unlikely/unlikely/neutral)	157 (32.2)	203 (27.4)		
Yes (likely/very likely)	330 (67.8)	538 (72.6)	0.07	0.20
**Illness perceptions related to sexually transmitted infections (STI)**				
BIPQ related to STI, mean (SD)				
Identity	7.7 (2.3)	7.8 (2.2)	0.58	0.52
Timeline	6.6 (2.6)	6.8 (2.6)	0.08	0.29
Personal control	5.8 (2.4)	5.9 (2.4)	0.56	0.22
Treatment control	7.5 (2.1)	7.5 (2.1)	0.47	0.79
Consequences	7.2 (2.2)	7.1 (2.0)	0.83	0.16
Concern	7.7 (2.3)	7.9 (2.3)	0.32	0.71
Coherence	5.1 (2.0)	4.8 (2.0)	0.003	0.001
Emotions	7.4 (2.2)	7.8 (2.1)	0.001	0.06
**Perceptions related to HPV DNA testing in general based on the Health Belief Model**				
Perceived benefits of HPV DNA testing, agree/strongly agree				
HPV DNA testing could detect HPV infection earlier, so as to have better treatment outcomes	404 (83.0)	664 (89.6)	0.001	0.06
HPV DNA testing could prevent cancers caused by HPV infection	387 (79.5)	576 (77.7)	0.47	0.56
Perceived Benefit Scale ^a^, mean (SD)	8.4 (1.4)	8.3 (1.6)	0.82	0.41
Perceived barriers to receive HPV DNA testing, agree/strongly agree				
Others would think you are having high-risk sexual behaviors	177 (36.3)	115 (15.5)	< 0.001	< 0.001
HPV DNA testing would cause pain and discomfort	65 (13.3)	262 (35.4)	< 0.001	< 0.001
Perceived Barrier Scale ^b^, mean (SD)	6.1 (1.4)	5.7 (1.4)	< 0.001	< 0.001
People who are important to you suggest that you take up HPV DNA testing (cue to action)				
Strongly disagree/disagree/neutral	190 (39.0)	284 (38.3)		
Agree/strongly agree	297 (61.0)	457 (61.7)	0.81	0.25
It is easy for you to take up HPV DNA testing in the next year if you want to (perceived self-efficacy)				
Strongly disagree/disagree/neutral	204 (41.9)	400 (40.5)		
Agree/strongly agree	283 (58.1)	441 (59.5)	0.63	0.98
**Perceptions specific to self-collected samples for HPV DNA testing**				
Perceived benefits specific to self-collected samples for HPV DNA testing, agree/strongly agree				
Performing self-collected samples for HPV DNA testing is easy for you	229 (47.0)	223 (30.1)	< 0.001	< 0.001
Performing self-collected samples for HPV DNA testing is very convenient	239 (49.1)	310 (41.8)	0.01	0.03
Using self-collected samples for HPV DNA testing could reduce risk of SARS-CoV-2 infection during the pandemic	247 (50.7)	399 (53.8)	0.28	0.20
Using self-collected samples for HPV DNA testing can avoid being stigmatized by testing service providers	235 (48.3)	236 (31.8)	< 0.001	< 0.001
Perceived Benefit Specific to Self-collected Samples for HPV DNA Testing Scale ^c^, mean (SD)	14.2 (2.9)	13.1 (2.9)	< 0.001	< 0.001
You are concerned about the accuracy of self-collected samples for HPV DNA testing (perceived barrier)				
Strongly disagree/disagree/neutral	351 (72.1)	387 (52.2)		
Agree/strongly agree	136 (27.9)	354 (47.8)	< 0.001	< 0.001
People who are important to you suggest that you take up self-collected samples for HPV DNA testing (cue to action)				
Strongly disagree/disagree/neutral	185 (38.0)	305 (41.2)		
Agree/strongly agree	302 (62.0)	436 (58.8)	0.27	0.02
It is easy for you to take up self-collected samples for HPV DNA testing in the next year if you want to (perceived self-efficacy)				
Strongly disagree/disagree/neutral	176 (36.1)	303 (40.9)		
Agree/strongly agree	311 (63.9)	438 (59.1)	0.10	0.06

Adjusted *P* values: adjusted for background variables with *p* < 0.05 in between-group comparison

^a^Perceived Benefit Scale, 2 items, Cronbach’s alpha: 0.69; one factor was identified by exploratory factor analysis, explaining for 76.6% of total variance

^b^Perceived Barrier Scale, 2 items, Cronbach’s alpha: 0.73; one factor was identified by exploratory factor analysis, explaining for 57.6% of total variance

^c^Perceived Benefit Specific to Self-collected Samples for HPV DNA Testing Scale, 4 items, Cronbach’s alpha: 0.69; one factor was identified by exploratory factor analysis, explaining for 55.6% of total variance

### Illness representation of STI and perceptions related to HPV DNA testing in general and self-collected samples for HPV DNA testing

As compared to females, heterosexual males scored higher in the coherence of STI (mean [SD]: 5.1 [2.0] versus 4.8 [2.0], *p* = 0.001), perceived more barriers to receive HPV DNA testing in general (mean [SD]: 6.1 [1.4] versus 5.7 [1.4], *p* < 0.001), and perceived higher benefits (mean [SD]: 14.2 [2.9] versus 13.1 [2.9], *p* < 0.001) and cue to action related to self-collected samples for HPV DNA testing (62.0% versus 58.8%, *p* = 0.02), but less concern about the accuracy of self-collected samples for HPV DNA testing (27.9% versus 47.8%, *p* < 0.001) (Table 2).

### Factors associated with behavioral intention to take up free clinician-collected samples for HPV DNA testing

In bivariate analysis, use of HPV testing in lifetime, sexual intercourse with RP in the past year, and condomless sex with RP in the past year were associated with higher intention to take up free clinician-collected samples for HPV DNA testing in the next year among in both heterosexual males and females. History of genital warts, sexual intercourse with NRP and commercial sex partners, and condomless sex with NRP were correlated with higher behavioral intention among heterosexual males, but not among females. Higher education level and history of HPV infection and HPV vaccination were associated with the dependent variable among females, but not among heterosexual males (Table 3).

**Table 3:** Associations between background characteristics and behavioral intention to take up free clinician-collected and self-collected samples for HPV DNA testing among heterosexual male and female participants

	Behavioral intention to take up free clinician-collected samples for HPV DNA testing				Behavioral intention to take up free self-collected samples for HPV DNA testing			
	Heterosexual male (*n* = 487)		Female (*n* = 741)		Heterosexual male (*n* = 487)		Female (*n* = 741)	
	OR (95% CI)	*P* values	OR (95% CI)	*P* values	OR (95% CI)	*P* values	OR (95% CI)	*P* values
**Socio-demographic characteristics**								
Age group, years								
18–30	1.0		1.0		1.0		1.0	
31–40	1.38 (0.68, 2.78)	0.37	1.70 (0.99, 2.91)	0.05	0.76 (0.35, 1.68)	0.50	1.73 (1.04, 2.87)	0.04
41–50	0.96 (0.48, 1.94)	0.92	1.25 (0.70, 2.23)	0.45	0.55 (0.25, 1.21)	0.14	1.19 (0.69, 2.06)	0.53
51–60	1.17 (0.59, 2.32)	0.65	1.13 (0.63, 2.02)	0.68	0.67 (0.31, 1.45)	0.31	1.38 (0.79, 2.41)	0.27
> 60	1.04 (0.51, 2.11)	0.92	0.76 (0.41, 1.41)	0.39	0.49 (0.22, 1.07)	0.08	0.68 (0.38, 1.23)	0.20
Ethnicity								
Chinese	1.0		1.0		1.0		1.0	
Other ethnicities	1.83 (0.19, 17.72)	0.60	0.91 (0.24, 3.41)	0.89	1.43 (0.15, 13.87)	0.76	0.75 (0.22, 2.52)	0.64
Marital status								
Currently single/divorced/widowed	1.0		1.0		1.0		1.0	
Married	0.89 (0.60, 1.31)	0.54	1.35 (0.95, 1.90)	0.09	0.75 (0.50, 1.13)	0.16	1.18 (0.85, 1.64)	0.33
Education level								
Primary or below	1.0		1.0		1.0		1.0	
Secondary	0.92 (0.29, 2.95)	0.89	2.41 (1.09, 5.29)	0.03	0.90 (0.28, 2.87)	0.86	2.05 (0.95, 4.45)	0.07
Diploma or associate degrees	1.48 (0.43, 5.11)	0.54	2.19 (0.94, 5.10)	0.07	3.38 (0.92, 12.45)	0.07	2.40 (1.04, 5.53)	0.04
University or above	1.01 (0.32, 3.18)	0.99	2.22 (1.02, 4.80)	0.04	1.40 (0.44, 4.41)	0.57	2.03 (0.95, 4.35)	0.07
Employment status								
Full time	1.0		1.0		1.0		1.0	
Part-time/self-employed/unemployed/retired/student/housewife	0.87 (0.58, 1.32)	0.52	0.80 (0.56, 1.13)	0.20	0.72 (0.47, 1.09)	0.12	0.78 (0.56, 1.09)	0.14
Monthly personal income, HK$ (US$)								
< 15,000 (1923)	1.0		1.0		1.0		1.0	
15,000–30,000 (1923–3846)	1.37 (0.75, 2.51)	0.31	1.05 (0.65, 1.69)	0.85	1.67 (0.90, 3.11)	0.10	1.13 (0.71, 1.81)	0.61
30,001–50,000 (3847–6410)	0.89 (0.47, 1.66)	0.71	1.12 (0.63, 2.01)	0.70	1.25 (0.66, 2.39)	0.50	0.94 (0.54, 1.63)	0.83
> 50,000 (6410)	0.97 (0.50, 1.90)	0.94	1.74 (0.79, 3.85)	0.17	1.21 (0.61, 2.40)	0.60	1.26 (0.62, 2.56)	0.52
Refuse to disclose	0.63 (0.32, 1.21)	0.16	0.81 (0.50, 1.30)	0.38	0.68 (0.35, 1.32)	0.25	0.65 (0.41, 1.03)	0.07
Living alone								
No	1.0		1.0		1.0		1.0	
Yes	1.28 (0.69, 2.40)	0.44	0.84 (0.44, 1.58)	0.58	1.97 (0.96, 4.06)	0.07	0.79 (0.43, 1.43)	0.43
Having medical insurance								
No	1.0		1.0		1.0		1.0	
Yes	1.06 (0.71, 1.58)	0.77	1.08 (0.76, 1.55)	0.67	1.46 (0.97, 2.19)	0.07	1.11 (0.79, 1.55)	0.56
**Health conditions**								
Presence of any chronic diseases								
No	1.0		1.0		1.0		1.0	
Yes	1.04 (0.67, 1.62)	0.86	0.79 (0.48, 1.30)	0.36	0.89 (0.57, 1.40)	0.61	1.01 (0.62, 1.65)	0.96
History of HPV infection								
No	1.0		1.0		1.0		1.0	
Yes	N.A	N.A	4.18 (1.28, 13.69)	0.02	N.A	N.A	2.93 (1.13, 7.55)	0.03
History of genital warts								
No	1.0		1.0		1.0		1.0	
Yes	10.88 (1.44, 82.44)	0.02	N.A	N.A	8.47 (1.12, 64.25)	0.04	N.A	N.A
**History of HPV testing and HPV vaccination**								
Ever had Pap smear testing experience								
No			1.0				1.0	
Yes	N.A	N.A	1.27 (0.87, 1.85)	0.22	N.A	N.A	1.21 (0.84, 1.75)	0.30
Use of HPV testing in the lifetime								
No	1.0		1.0		1.0		1.0	
Yes	4.72 (1.39, 16.01)	0.01	2.38 (1.46, 3.86)	< 0.001	2.60 (0.88, 7.71)	0.09	1.65 (1.09, 2.50)	0.02
History of HPV vaccination								
No	1.0		1.0		1.0		1.0	
Yes	2.84 (0.95, 8.53)	0.06	1.76 (1.15, 2.69)	0.01	2.21 (0.73, 6.63)	0.16	1.57 (1.06, 2.32)	0.02
**Sexual behaviors in the past year**								
Sexual intercourse with regular sex partners (RP)								
No	1.0		1.0		1.0		1.0	
Yes	1.62 (1.10, 2.37)	0.01	1.77 (1.22, 2.57)	0.002	1.78 (1.19, 2.66)	0.01	1.49 (1.06, 2.10)	0.02
Sexual intercourse with non-regular sex partners (NRP)								
No	1.0		1.0		1.0		1.0	
Yes	5.90 (1.77, 19.74)	0.004	0.96 (0.38, 2.45)	0.94	1.97 (0.79, 4.93)	0.15	1.20 (0.47, 3.05)	0.70
Sexual intercourse with commercial sex partners								
No	1.0		1.0		1.0		1.0	
Yes	4.68 (1.80, 12.16)	0.001	N.A	N.A	2.89 (1.19, 7.04)	0.02	0.38 (0.02, 6.04)	0.49
Condomless sex with RP								
No	1.0		1.0		1.0		1.0	
Yes	1.79 (1.18, 2.70)	0.01	1.56 (1.04, 2.35)	0.03	1.76 (1.14, 2.71)	0.01	1.67 (1.13, 2.47)	0.01
Condomless sex with NRP								
No	1.0		1.0		1.0		1.0	
Yes	6.09 (1.40, 26.45)	0.02	1.23 (0.40, 3.71)	0.72	2.96 (0.86, 10.21)	0.09	1.53 (0.50, 4.62)	0.46
Condomless sex with commercial sex partners								
No	1.0		1.0		1.0		1.0	
Yes	5.60 (0.70, 44.58)	0.10	N.A	N.A	1.93 (0.40, 9.18)	0.41	N.A	N.A

*OR* crude odds ratios

*CI* confidence interval

*N.A.* not applicable

After adjusting for these significant background characteristics, perceived existing treatment could control STI (treatment control) (AOR: 1.23, 95% CI: 1.12, 1.36, *p* < 0.001 and AOR: 1.11, 95% CI: 1.02, 1.20, *p* = 0.02), more concerned about STI (concern) (AOR: 1.20, 95% CI: 1.10, 1.31, *p* < 0.001 and AOR: 1.12, 95% CI: 1.04, 1.20, *p* = 0.003), perceived more benefits of HPV testing in general (AOR: 1.84, 95% CI: 1.56, 2.15, *p* < 0.001 and AOR: 1.45, 95% CI: 1.32, 1.65, *p* < 0.001), agreed that significant others would suggest them take up HPV testing (cue to action) (AOR: 2.06, 95% CI: 1.39, 3.05, *p* < 0.001 and AOR: 1.82, 95% CI: 1.27, 2.60, *p* = 0.001), and perceived self-efficacy to take up HPV testing (AOR: 4.61, 95% CI: 3.06, 6.93, *p* < 0.001 and AOR: 2.67, 95% CI: 1.86, 3.84, *p* < 0.001) were positively associated with behavioral intention among both heterosexual males and females. In addition, heterosexual males who perceived more severe symptoms if contracted STI (identity) (AOR: 1.16, 95% CI: 1.07, 1.26, *p* = 0.001), longer duration of STI (timeline) (AOR: 1.11, 95% CI: 1.02, 1.19, *p* = 0.01), more negative effects of STI on their lives (consequences) (AOR: 1.13, 95% CI: 1.03, 1.23, *p* = 0.01), more understanding of STI (coherence) (AOR: 1.12, 95% CI: 1.01, 1.24, *p* = 0.03), and stronger negative emotions if contracted STI (emotions) (AOR: 1.16, 95% CI: 1.06, 1.27, *p* = 0.001) were having higher behavioral intention to take up clinician-collected samples for HPV DNA testing (Table 4).

**Table 4:** Factors associated with behavioral intention to take up free clinician-collected and self-collected samples for HPV DNA testing among heterosexual male and female participants

	Behavioral intention to take up free clinician-collected samples for HPV DNA testing				Behavioral intention to take up free self-collected samples for HPV DNA testing			
	Heterosexual male (*n* = 487)		Female (*n* = 741)		Heterosexual male (*n* = 487)		Female (*n* = 741)	
	AOR (95% CI)	*P* values	AOR (95% CI)	*P* values	AOR (95% CI)	*P* values	AOR (95% CI)	*P* values
**Illness perceptions related to sexually transmitted infections (STI)**								
BIPQ related to STI, mean (SD)								
Identity	1.16 (1.07, 1.26)	0.001	1.03 (0.95, 1.11)	0.48	1.15 (1.06, 1.25)	0.001	1.02 (0.95, 1.10)	0.65
Timeline	1.11 (1.02, 1.19)	0.01	1.01 (0.94, 1.08)	0.82	1.08 (1.00, 1.15)	0.06	1.02 (0.95, 1.08)	0.61
Personal control	1.02 (0.94, 1.10)	0.73	0.99 (0.92, 1.07)	0.86	1.00 (0.92, 1.08)	0.96	0.96 (0.89, 1.02)	0.20
Treatment control	1.23 (1.12, 1.36)	< 0.001	1.11 (1.02, 1.20)	0.02	1.17 (1.06, 1.28)	0.001	1.07 (0.99, 1.16)	0.09
Consequences	1.13 (1.03, 1.23)	0.01	1.00 (0.92, 1.09)	0.97	1.14 (1.04, 1.25)	0.004	1.02 (0.94, 1.11)	0.67
Concern	1.20 (1.10, 1.31)	< 0.001	1.12 (1.04, 1.20)	0.003	1.18 (1.08, 1.28)	< 0.001	1.08 (1.01, 1.16)	0.03
Coherence	1.12 (1.01, 1.24)	0.03	0.95 (0.87, 1.04)	0.30	1.04 (0.94, 1.14)	0.48	0.99 (0.91, 1.08)	0.81
Emotions	1.16 (1.06, 1.27)	0.001	1.08 (0.99, 1.17)	0.09	1.15 (1.05, 1.25)	0.003	1.06 (0.98, 1.15)	0.16
**Perceptions related to HPV DNA testing in general based on the Health Belief Model**								
Perceived Benefit Scale	1.84 (1.56, 2.15)	< 0.001	1.45 (1.32, 1.65)	< 0.001	1.80 (1.54, 2.10)	< 0.001	1.54 (1.37, 1.73)	< 0.001
Perceived Barrier Scale	0.87 (0.76, 1.01)	0.06	0.91 (0.80, 1.03)	0.12	0.92 (0.80, 1.06)	0.25	1.00 (0.89, 1.13)	0.97
People who are important to you suggest that you take up HPV DNA testing (cue to action)								
Strongly disagree/disagree/neutral	1.0		1.0		1.0		1.0	
Agree/strongly agree	2.06 (1.39, 3.05)	< 0.001	1.82 (1.27, 2.60)	0.001	2.44 (1.64, 3.64)	< 0.001	2.14 (1.52, 3.02)	< 0.001
It is easy for you to take up HPV DNA testing in the next year if you want to (perceived self-efficacy)								
Strongly disagree/disagree/neutral	1.0		1.0		1.0		1.0	
Agree/strongly agree	4.61 (3.06, 6.93)	< 0.001	2.67 (1.86, 3.84)	< 0.001	3.59 (2.39, 5.38)	< 0.001	2.82 (1.99, 4.01)	< 0.001
**Perceptions specific to self-collected samples for HPV DNA testing**								
Perceived Benefit Specific to Self-collected Samples for HPV DNA Testing Scale	N.A	N.A	N.A	N.A	1.32 (1.22, 1.44)	< 0.001	1.35 (1.25, 1.45)	< 0.001
You are concerned about the accuracy of self-collected samples for HPV DNA testing (perceived barrier)								
Strongly disagree/disagree/neutral					1.0		1.0	
Agree/strongly agree	N.A	N.A	N.A	N.A	1.13 (0.73, 1.75)	0.58	0.75 (0.54, 1.06)	0.10
People who are important to you suggest that you take up self-collected samples for HPV DNA testing (cue to action)								
Strongly disagree/disagree/neutral					1.0		1.0	
Agree/strongly agree	N.A	N.A	N.A	N.A	2.27 (1.52, 3.37)	< 0.001	3.08 (2.16, 4.40)	< 0.001
It is easy for you to take up self-collected samples for HPV DNA testing in the next year if you want to (perceived self-efficacy)								
Strongly disagree/disagree/neutral					1.0		1.0	
Agree/strongly agree	N.A	N.A	N.A	N.A	3.63 (2.41, 5.47)	< 0.001	5.48 (3.76, 7.99)	< 0.001

*OR* crude odds ratios

*AOR* adjusted odds ratios, odds ratios adjusted for significant background characteristics listed in Table 3

*CI* confidence interval

*N.A*. not applicable

### Factors associated with behavioral intention to take up free self-collected samples for HPV DNA testing

Sexual intercourse with RP and condomless sex with such partner in the past year was associated with higher intention to take up self-collected samples for HPV DNA testing among both heterosexual males and females. Females who were 31–40 years, had higher education, and with a history of HPV testing and HPV vaccination also had higher intention to take up self-collected samples for HPV DNA testing. In addition, a history of genital warts and sexual intercourse with commercial sex partners was positively associated with behavioral intention to take up self-collected samples for HPV DNA testing among heterosexual males (Table 3).

After adjusting for these significant background characteristics, more concerned about STI (concern) (AOR: 1.18, 95% CI: 1.08, 1.28, *p* < 0.001 and AOR: 1.08, 95% CI: 1.01, 1.16, *p* = 0.03), perceived benefits of HPV DNA testing in general (AOR: 1.80, 95% CI: 1.54, 2.10, *p* < 0.001 and AOR: 1.54, 95% CI: 1.37, 1.73, *p* < 0.001) and self-collected samples for HPV DNA testing (AOR: 1.32, 95% CI: 1.22, 1.44, *p* < 0.001 and AOR: 1.35, 95% CI: 1.25, 1.45, *p* < 0.001), cue to action related to HPV DNA testing in general (AOR: 2.44, 95% CI: 1.64, 3.64, *p* < 0.001 and AOR: 2.14, 95% CI: 1.52, 3.02, *p* = 0.001) and self-collected samples for HPV DNA testing (AOR: 2.27, 95% CI: 1.52, 3.37, *p* < 0.001 and AOR: 3.08, 95% CI: 2.16, 4.40, *p* < 0.001), and perceived self-efficacy to take up HPV DNA testing in general (AOR: 3.59, 95% CI: 2.39, 5.38, *p* < 0.001 and AOR: 2.82, 95% CI: 1.99, 4.01, *p* < 0.001) and self-collected samples for HPV DNA testing (AOR: 3.63, 95% CI: 2.41, 5.47, *p* < 0.001 and AOR: 5.48, 95% CI: 3.76, 7.99, *p* < 0.001) were positively associated with behavioral intention among both heterosexual males and females. In addition, heterosexual males who perceived more severe symptoms if contracted STI (identity) (AOR: 1.15, 95% CI: 1.06, 1.25, *p* = 0.001), existing treatment could control STI (treatment control) (AOR: 1.17, 95% CI: 1.06, 1.28, *p* = 0.001), more negative effects of STI on their lives (consequences) (AOR: 1.14, 95% CI: 1.04, 1.25, *p* = 0.004), and stronger negative emotions if contracted STI (emotions) (AOR: 1.15, 95% CI: 1.05, 1.25, *p* = 0.001) were having higher behavioral intention to take up clinician-collected samples for HPV DNA testing (Table 4).

## Discussion

This is one of the first studies comparing levels of behavioral intention to receive different types of HPV tests and their determinants between heterosexual males and females. This study was based on a territory-wide household survey with a relatively large sample size. Our findings extended the application of illness representation and the HBM in explaining health behaviors. Our study provided a knowledge basis to inform service planning and the development of tailored interventions promoting HPV DNA testing uptake for females. It also provided data on the acceptability of HPV DNA testing for males. Over 70% of the female participants used cytology testing in their lifetime, which met the WHO recommendation of HPV screening rate for females. However, relatively few females (22.7%) received HPV testing. Such uptake rate of HPV testing was lower than that of females in other places [25–29]. Only 5% of the heterosexual males had ever used HPV testing, which was much lower than their female counterparts. It is likely that some heterosexual males with high-risk HPV infection are not identified. Monitoring HPV infection status might facilitate heterosexual males to adopt preventive measures (e.g., consistent condom use, taking up HPV vaccination) to reduce the risk of transmitting HPV to their female sex partners.

Over three-quarters of females intended to receive free clinician-collected samples for HPV DNA testing in the next year. Such level of behavioral intention was higher than those observed among females in other countries (45.6–69.7%) [30, 31]. Fewer heterosexual males intended to receive clinician-collected samples for HPV DNA testing compared to females. Lower awareness of HPV DNA testing among heterosexual males than females might partially contribute to the difference. Self-collected samples for HPV DNA testing, if provided for free, may be an alternative option to increase HPV screening coverage in future, as the level of behavioral intention to receive such testing was equally high among heterosexual males and females.

Similar to previous studies, heterosexual males and females with a history of HPV infection and/or genital warts had a higher intention to receive clinician-collected and/or self-collected samples for HPV testing [25, 29–31, 34]. In Hong Kong SAR, women with prior HPV infection are recommended to receive frequent screening based on the doctors’ assessment [16]. In addition, heterosexual males and females with such a history of infection were more likely to consider HPV as a health threat. Sexual intercourse and condomless sex with RP were associated with higher behavioral intention to receive both clinician-collected and self-collected samples for HPV DNA testing among heterosexual males and females. Protecting RP against HPV infection might be a motivation for them to receive such testing. As compared to females, heterosexual males were more likely to report sexual intercourse with NRP and commercial sex partners in the past six months. Having sex with such partners was associated with a higher intention to receive both types of HPV DNA testing among heterosexual males. It was possible that heterosexual males with such sexual risk behaviors perceived a higher risk of HPV-related diseases and hence a stronger need to receive HPV testing. Health promotion to increase HPV DNA testing uptake should give more attention to females with lower education, as they reported lower intention to receive both types of HPV DNA testing. Such finding was consistent with previous studies [25, 29–31, 34].

Our findings highlighted the impacts of illness representations of STI on the decision to receive HPV DNA testing. The associations between different domains of illness representation and behavioral intention were different between these two populations. Seven out of eight domains of illness representation were associated with behavioral intention among males. While only treatment control and concern were associated with behavioral intention among females. Such findings suggested that heterosexual males might consider taking up HPV DNA testing as a coping strategy for STI. Since cervical cancer is a larger health threat than STI perceived by females [47], illness representation of STI had smaller impacts on their HPV testing behaviors. We found that heterosexual males who perceived more negative impacts of STI on their daily lives (consequences), expected more severe symptoms after contracting STI (identity), longer duration of STI (timeline), and perceived treatment could better control STI (treatment control) had higher intention to receive both types of HPV DNA testing. They might consider HPV DNA testing as a means for earlier HPV infection detection and treatment to prevent severe consequences. It is evident that STI would bring concerns and negative emotions among heterosexual males, and such concerns and negative emotions were associated with higher intention to receive both types of HPV testing. It is possible to guide those with high levels of concerns and negative emotions to receive HPV testing as a positive coping response, which transfers concerns and negative emotions into preventive behaviors. Consistent with studies among females which found higher knowledge of HPV as a facilitator of HPV testing uptake, better coherence of STI was associated with higher intention to receive clinician-collected HPV testing among heterosexual males [25, 29–31, 34].

The HBM is a potentially useful framework to explain behaviors related to clinician-collected and self-collected samples for HPV DNA testing. The associations between HBM constructs and behavioral intention to receive both types of HPV DNA testing were similar between heterosexual males and females. A high proportion of heterosexual males and females perceived some benefits of HPV testing in general, and such perceptions were associated with higher intention to receive clinician-collected samples for HPV testing. Future health promotion campaigns should strengthen these perceived benefits (e.g., early detection and timely treatment for HPV and cancers) among both populations. Perceived cue to action and self-efficacy related to HPV testing in general were both facilitators to receive clinician-collected samples for HPV testing. Future programs should involve significant others (e.g., spouse, family members, and doctors) to give reminders to take up HPV testing as a strong cue to action. Facilitating people to form an action plan to receive HPV testing is a potentially useful strategy to strengthen perceived self-efficacy [48]. The aforementioned strategies may also be useful to promote self-collected samples for HPV testing among both heterosexual males and females. In addition, future campaigns promoting self-collected HPV testing should also emphasize its benefits (e.g., easy to use, convenient, less stigma originated from service providers), as perceived benefit was a facilitator. Having significant others providing reminders and facilitating action planning to receive self-collected samples for HPV testing are also potentially useful health promotion strategies.

This study had several limitations. First, the response rate was relatively low, but was similar to similar postal surveys conducted in Hong Kong and other regions [49]. As compared to the census data, older adults (those aged 60 years or above) were under-sampled by this study [50]. It was possible that older adults found the contents of this survey (STI and HPV DNA testing) less relevant to them and hence did not respond to the invitation. Second, we were not able to collect information from those who refused to join the study and hence could not compare the characteristics between refusals and participants. Selection bias existed. Third, participants might over-report behavioral intention to receive HPV testing and under-report sexual risk behaviors or history of HPV infection due to social desirability. We were not able to verify the responses. Fourth, our outcomes were behavioral intentions instead of actual behaviors. Behavioral intention is a strong predictor of actual behaviors. Fifth, the Cronbach alphas of the scales used in this study was relatively low (0.69–0.73), which might be due to the low number of questions in the scale. Moreover, the cross-sectional study design could not establish causal relationships.

## Conclusions

HPV DNA testing was under-utilized in Hong Kong. Free self-collected samples for HPV testing were highly acceptable by both heterosexual males and females. Illness representation of STI and the HBM could explain behaviors related to HPV DNA testing.

## Data Availability

The datasets generated and/or analyzed during the current study are not publicly available as they contain sensitive personal behaviors but are available from the corresponding author upon reasonable request.

## Abbreviations

HPV: Human papillomavirus
WHO: World Health Organization
MSM: Men who have sex with men
STI: Sexually transmitted infections
QR: Quick response
RP: Regular sex partner
NRP: Non-regular sex partner
B-IPQ: Brief Illness Perception Questionnaire
HBM: Health Belief Model
OR: Crude odds ratio
AOR: Adjusted odds ratios
CI: Confidence interval
SD: Standard deviation
